# Creatures of habit: accounting for the role of habit in implementation research on clinical behaviour change

**DOI:** 10.1186/1748-5908-7-53

**Published:** 2012-06-09

**Authors:** Per Nilsen, Kerstin Roback, Anders Broström, Per-Erik Ellström

**Affiliations:** 1 Division of Health Care Analysis, Department of Medicine and Health, Linköping University, SE-581 83, Linköping, Sweden; 2 Department of Nursing Science, School of Health Sciences, Jönköping University, Jönköping, Sweden; 3 Department of Clinical Neurophysiology, Linköping University Hospital, SE-581 85, Linköping, Sweden; 4 HELIX VINN Excellence Centre, Linköping University, SE-581 83, Linköping, Sweden

**Keywords:** Habits, Social cognitive theories, Clinical behaviour, Interventions

## Abstract

**Background:**

Social cognitive theories on behaviour change are increasingly being used to understand and predict healthcare professionals’ intentions and clinical behaviours. Although these theories offer important insights into how new behaviours are initiated, they provide an incomplete account of how changes in clinical practice occur by failing to consider the role of cue-contingent habits. This article contributes to better understanding of the role of habits in clinical practice and how improved effectiveness of behavioural strategies in implementation research might be achieved.

**Discussion:**

Habit is behaviour that has been repeated until it has become more or less automatic, enacted without purposeful thinking, largely without any sense of awareness. The process of forming habits occurs through a gradual shift in cognitive control from intentional to automatic processes. As behaviour is repeated in the same context, the control of behaviour gradually shifts from being internally guided (*e.g.*, beliefs, attitudes, and intention) to being triggered by situational or contextual cues. Much clinical practice occurs in stable healthcare contexts and can be assumed to be habitual. Empirical findings in various fields suggest that behaviours that are repeated in constant contexts are difficult to change. Hence, interventions that focus on changing the context that maintains those habits have a greater probability of success. Some sort of contextual disturbance provides a window of opportunity in which a behaviour is more likely to be deliberately considered. Forming desired habits requires behaviour to be carried out repeatedly in the presence of the same contextual cues.

**Summary:**

Social cognitive theories provide insight into how humans analytically process information and carefully plan actions, but their utility is more limited when it comes to explaining repeated behaviours that do not require such an ongoing contemplative decisional process. However, despite a growing interest in applying behavioural theory in interventions to change clinical practice, the potential importance of habit has not been explored in implementation research.

## Introduction

Implementation interventions to modify clinical practice typically show small to modest effects on healthcare professionals’ behaviour [1,2]. The limited success of interventions to change clinical practice has been attributed to failure to properly apply behavioural theory to intervention design [3-5]. However, in recent years, there has been considerable interest in the use of behavioural theory to better understand barriers to implementation, inform the design of interventions to change clinical practice, and explore mediating mechanisms and potential moderators of such interventions.

Social cognitive theories on behaviour change have been widely applied to understand why people in general do or do not adopt a given behaviour. These theories are increasingly being used in implementation research; it is argued that the determinants for healthcare professionals’ intentions and behaviours are similar to those concerning people in general. After all, it is usually the individual healthcare professional who decides whether or not to perform a specific clinical behaviour, such as prescribing an antibiotic for a sore throat, adhering to a hygiene recommendation, conducting a treatment follow-up, or providing advice on alcohol consumption.

This article highlights the lack of consideration of the role of habit in social cognitive theories used in implementation research. Habits are automatic responses to contextual cues, acquired through repetition of behaviour in the presence of these cues [6-9]. We argue that habit is a critical variable in understanding clinical practice change because healthcare professionals’ daily practice is predominantly habitual and therefore difficult to change through many conventional implementation interventions. The overall aim is to contribute to better understanding of the role of habits in clinical practice and how improved effectiveness of behavioural strategies in implementation research might be achieved.

### Social cognitive theories and habits

Social cognitive theories are the dominant theories used in clinical behaviour change research. The Theory of Reasoned Action [10], the Social Cognitive Theory [11], the Theory of Interpersonal Behaviour [12], and the Theory of Planned Behaviour [13] have been applied to identify variables that underlie healthcare professionals’ behaviours and to predict behaviour change. A systematic review by Godin *et al*. [5] identified 76 studies that used social cognitive theories to understand and predict healthcare professionals’ intentions and behaviours. The most commonly used theories were Theory of Reasoned Action and Theory of Planned Behaviour (essentially an extension of the Theory of Reasoned Action). Godin *et al.*[5] concluded that the Theory of Planned Behaviour was the most appropriate theory to predict behaviour, whereas other theories better captured factors that have an impact on behavioural intention.

Many social cognitive theories view intention as the key predictor of behavioural enactment. Intention refers to an individual’s motivation concerning the performance of a given behaviour. Research on population and patient behaviour change suggests that intention is a relatively good indicator of behaviour in longitudinal studies, but generally leaves 50 % to 60 % of the variance in behaviour unexplained [14]. Two systematic reviews [5,15] of the relationship between intention and clinical behaviour found only 16 studies, but suggested that the nature of the relationship was similar to that of non-healthcare professionals. The strength of the intention–behaviour relationship was found to vary depending on factors such as the specific clinical behaviour under study, professional category and whether self-reported or observational measures were used [5].

Overall, findings in different fields indicate a rather substantial intention–behaviour gap. Although someone with strong intentions to perform a given behaviour is more likely to enact the behaviour in question than someone with weak intentions, many people with strong motivation do not behave in accordance with this intention [14]. Numerous studies in various behavioural domains (including blood donation, seat belt use, choice of travel mode, and fast food purchase) have shown that the connection between intention and future behaviour is moderated by the degree to which the current behaviour is habitual; the utility of intention as a predictor of behaviour declines as habit strength increases [16-18].

Inherent in many social cognitive theories is the assumption that intention and hence behaviour can be influenced by the provision of appropriate information concerning a behaviour. Commonly used interventions in implementation research include dissemination of printed materials, such as guideline recommendations and various forms of education, including continuing medical education and educational outreach [4]. However, a growing body of research shows that individuals who have developed habitual behaviours become less likely to act on new information and may even avoid information that challenges the present behaviour [18]. People appear to form expectations for certain outcomes as behaviours become habitual, something that tends to reduce their sensitivity to change in a context that might otherwise result in new behaviour; people may overlook alternatives simply because their ongoing expectations reduce their awareness of new information [19-21]. Essentially, habits yield tunnel vision, thus reducing the effectiveness of interventions aimed at changing behaviour through conscious cognitive deliberation.

Social cognitive theories can offer insight into how humans analytically process information and carefully plan actions, but their utility is more limited when it comes to explaining repeated behaviours that do not require such an ongoing, contemplative decisional process [22]. Research on consumer habits suggests that most everyday activities are behaviours that we repeat over and over again. It has been estimated that approximately 45 % of everyday behaviours tend to be repeated in the same physical location every day [23]. In familiar and unvarying settings, behaviour tends to be guided more by habit than intention, but in novel or changing contexts behaviour will be regulated by intention; where habit and intention conflict, behaviour is more likely to proceed in line with habit than intention [24].

### Understanding habit

Habits have been studied extensively in the behaviourist tradition. Behaviourists consider habit as repeated behaviour that is established through learning. The behaviourist view posits a direct relationship between situation (stimulus) and behaviour (response). Explicit rewards are required after a response to a situational cue. The probability that a situation will elicit a behaviour depends on the frequency of performing the behaviour in the situation [25-28].

However, the conceptualization of habit strength as frequency of past behaviour is increasingly being disputed. Some behaviours turn quickly into habits, whereas others may require painstaking practice and frequent repetition. And although habitual behaviour may be frequently performed, frequent behaviour is not necessarily habitual [23,29,30]. Thus, behavioural recurrence does not constitute direct evidence for habitual behaviour. There is also recognition that rewards are not necessary for habit formation when a behaviour is intrinsically rewarding. In fact, providing external rewards can reduce intrinsic motivation to continue to perform a behaviour, thus hindering the habit formation process [31].

A new conceptualization of habit is emerging that differs from behaviourism by going beyond habit as merely repeated behaviour. More attention is paid to the automaticity aspect of habitual behaviour. There is general agreement that habits are automatic in the sense that they are enacted without purposeful thinking, largely without any sense of awareness, and can be performed quickly in parallel with other activities [23]. While habits form through repeated performance in unvarying settings, and many habits are performed frequently, several researchers consider that, once formed, the ‘active ingredient’ of habit is automaticity. This viewpoint presents behaviour frequency as a precursor and possible consequence of automaticity, rather than a part of the construct habit itself [9,32].

Definitions of habit that account for an automaticity dimension are consistent with so-called dual processing models, which distinguish between two distinct modes of reasoning and information processing. The automatic mode is typically described as fast, intuitive, and heuristic, whereas the intentional mode is characterized as rational, deliberate, slow, rule-based, and explicit. The models posit that the two processes are in constant operation as humans reason [33-35]. Sladek *et al*. [36] suggested that dual processing models can be applied for improved identification of factors that influence the uptake of new evidence by physicians.

### Forming and breaking habits

The process of forming habits occurs through a gradual shift in cognitive control from intentional to automatic processes. When a behaviour is performed many times, humans begin to use a sort of heuristic decision-making strategy, *i.e.*, a cognitive shortcut whereby we do not need to scrutinize all the consequences of enacting a certain behaviour [37]. As behaviour is repeated in the same context, the control of behaviour gradually shifts from being internally guided (*e.g.*, beliefs, attitudes, and intention) to being triggered by situational or contextual cues, such as automatically looking both ways before crossing the street, strapping on the seat belt when entering a car, or saying ‘amen’ at the end of a public prayer in church. Wood and Neal [7] describe this as ‘outsourcing’ of behavioural control to contextual cues. A shift from deliberate to automatic processes has been found across a broad range of social behaviours [38]. Provided that the context remains the same and the response satisfies the person’s objectives, these associations acquire a degree of automaticity [6]. The context is the environment in which behaviour takes place; the features or cues that trigger action can be anything from physical objects and preceding actions to geographical features or people [16].

Empirical findings in various fields suggest that behaviours that are repeated in constant contexts are difficult to change [39-41]. Hence, interventions that focus on changing the context that maintains those habits have a greater probability of success. Some sort of contextual disturbance provides a window of opportunity in which a behaviour is more likely to be deliberately considered. This effectively makes behaviour-relevant information more salient and influential [18,20,40]. This is clearly in line with Dewey’s [42] view of learning as a process that begins with a perceived difficulty or disturbance in a routinized task or situation. To some extent, it is also related to Heidegger’s [43] concept of unreadiness-to-hand, *i.e.*, an unpredicted disturbance that makes us recognize and see things we do not normally notice or have come to take for granted (presence-at-hand). The relative effectiveness of reminders (signals before or during performance of a behaviour) as a clinical behaviour change intervention [44] can be attributed to such a contextual disturbance mechanism [45].

Different types of interventions are needed to disrupt unwanted habits and/or to promote desired habits than those used to modify behaviour through conscious cognitive deliberation, as depicted by social cognitive theories. Because habits are triggered automatically in response to contextual cues, breaking ‘bad’ habits can be achieved by either removing persons from the environment that cues unwanted habitual responses or by modifying the context, *e.g.*, placing reminders in the environment. Research also suggests that vigilant monitoring can offer a means of inhibiting habits because it enables the individual to identify cues and exert control in order to reduce unwanted habitual responses [46]. Forming ‘good’ habits require behaviour to be carried out repeatedly in the presence of the same contextual cues. Recent research has shown that participants encouraged to perform a health-promoting behaviour regularly in familiar contexts achieved increases in habit-related automaticity, such that initial repetitions caused large increases in automaticity, but automaticity gains were reduced with each new repetition [8,47]. However, there is still a paucity of empirical research on habit formation techniques or interventions underpinned by habit theory.

### Addressing habits in clinical behaviour change research

There is an emerging recognition that habits might be a critical factor in explaining the difficulties of modifying clinical behaviour. Indeed, in a commentary Rochette *et al*. ([48]:1790) lamented that habit is still an ‘under-explored’ concept in research on clinical practice change because they believed it might help ‘explain the lack of uptake of new, scientifically sound practices’ by clinicians. Much clinical practice occurs in stable healthcare contexts and can be assumed to be habitual, thus making many clinical behaviours unlikely to be spontaneously reconsidered. For any intervention aimed at clinical behaviour change, we can only expect results if healthcare professionals have positive attitudes and a strong intention to modify the target behaviour. Positive attitudes and good intentions are not sufficient. Interventions may be successful in changing attitudes and intentions, but these changes are unlikely to be converted into the desired clinical behaviour if the specific behaviour that needs to be modified or removed is strongly habitual.

A range of behaviours have been described in clinical behaviour change research, including physical examination, ordering of laboratory tests, referral diagnosis, patient education, hand hygiene, and providing health promotion advice (*e.g.*, on alcohol use or physical activity). These behaviours are desirable, deemed necessary or recommended clinical actions according to guidelines or expert consensus [49]. It is likely that the non-adherence to many of these desired clinical behaviours is habitual for many healthcare professionals, although empirical research is lacking to establish the extent to which this is the case for various target behaviours. A few studies have also investigated actions that are undesirable, *e.g.*, prescribing antibiotic for a viral infection, thus being a potentially habitual behaviour that needs to be modified.

Two basic approaches have been used to account for habitual qualities in clinical practice. A few studies (*e.g.*, [50-52]) have applied the Theory of Interpersonal Behaviour, which includes frequency of past behaviour as a potential predictor of intentions and behaviours. This conceptualization of habit thus does not account for the automaticity aspects of habit. The importance of habit (as frequency of past behaviour) as a predictor for intentions and behaviours has differed between these studies.

The other approach (*e.g.*, [53-56]) has involved investigating evidence of habit with reference to the behaviourist Operant Learning Theory [57]. In these studies, the respondents were asked whether a particular behaviour was automatic or not, *e.g.*, managing patients without antibiotics, referring patients with back pain for an X-ray or placing preventive fissure sealants in young dental patients—‘when I see a patient, I automatically consider behaviour X.’ These studies also enquired whether this behaviour was ‘usual practice.’ Habit operationalized this way has been found to be an important predictor of the outcome measures, although the results have been inconsistent. This automaticity approach appears to operationalize the habit construct with more theoretical purity than attempts to merely measure behavioural frequency, yet the complexity of the habit concept can hardly be captured with two questions.

There have also been attempts to expand on existing theories and construct new approaches to account for the presumed habitual quality of much clinical practice. Godin *et al*. [5] have proposed an augmented version of the Theory of Planned Behaviour that included habit as an additional construct. They argued (Godin *et al*. [5:9]) in their systematic review of studies based on social cognitive theories that habits ought to be addressed in future research because ‘many of the behaviours performed by healthcare professionals could be categorized as habitual.’ However, they did not provide a definition of the concept of habit. Michie *et al*. [58] have developed the ‘behaviour change wheel’ as a method for characterizing and designing behaviour change interventions. Resembling dual processing models, their expanded view of motivation encompasses both analytical and automatic processes, the latter involving emotions and impulses that arise from associative learning of habitual behaviour.

Beyond research on clinical behaviour, the Self-Rated Habit Index (SRHI) is the most widely applied instrument to assess habit strength. It has been used with a variety of behaviours, including exercising, engaging in sports activity, watching television, eating unhealthy snacks, fruit consumption, and transportation mode choice. The SRHI is a 12-item scale that assesses the automaticity of a behaviour together with its antecedent (frequency of repetition) and possible consequences (including assimilation of the behaviour into one’s self-identity) [59]. A recent systematic review of 22 healthy eating and physical activity studies based on the SRHI found a medium-to-strong habit-behaviour correlation and a habit-intention trade-off, such that the impact of intentions on behaviour lessened as habit strength increased [24]. Concerns have been raised about validity aspects of the SRHI. For example, the self-identity aspect does not appear to be a necessary feature of the habit concept and the inclusion of repetition indicators may inflate the habit-behaviour correlation [9,28]. Further, the validity of self-reports of behaviours that are assumed to be automatic, *i.e.*, operating outside of awareness, may be questioned, as suggested by Sniehotta and Presseau [9], who have argued that a self-report of one’s habitual behaviour more likely reflects an inference of the consequences of the habit and these consequences may not always be salient. Challenges involved in operationalizing and measuring habit are a reason why habit measurement has not advanced further.

## Discussion

This article has pointed to the importance of integrating the habit concept into research on clinical behaviour change for better understanding of the difficulties of changing clinical practice and improved explanation as to the extent to which various non-best-practice clinical behaviours may be habitual. Understanding the target behaviours and barriers to change is an important precursor to the selection of interventions. There is a need to explore intervention strategies that account for the habitual nature of clinical practice by investigating various means of contextual changes or disturbances to break unwanted habits and/or achieving behavioural repetition in consistent contexts to promote formation of desired habits.

The widely used social cognitive theories offer valuable insight into how new behaviours are initiated, but they provide an incomplete account of how changes in clinical practice occur by failing to consider the critical role of habit. Healthcare professionals rarely weigh up the benefits and costs of a particular action in the precise, methodological way suggested by many of the social cognitive theories. However, despite the obvious potential of the habit concept as an explanatory factor, this issue has not been addressed much in research on clinical behaviour change.

Ultimately, the question of ‘why do healthcare professionals not implement and adhere to best practice?’ may be explained not by assuming that there is a problem with knowledge, skills, attitudes, or motivation, but by recognizing that all people, healthcare professionals included, are prone to developing efficient and automatically activated habits. Essentially, healthcare professionals are creatures of habit.

## Competing interests

The authors declare that they have no competing interests.

## Authors’ contributions

All authors contributed actively to this paper. PN had the original research idea and wrote the first draft, which was discussed with all co-authors, KR, AB, and P-EE. Further drafts were developed in close collaboration among all four authors. All authors approved the final version of the paper.
